# Dynamic characteristics of skin reaction force in different body postures

**DOI:** 10.1038/s41598-023-27489-4

**Published:** 2023-02-08

**Authors:** Nick Marsidi, Karlijn M. J. Scheepens, Jelle J. Goeman, Tim Horeman, Roel E. Genders

**Affiliations:** 1 Department of Dermatology, Leiden University Medical Center, Albinusdreef 2, 2333 ZA Leiden, The Netherlands; 2 Department of Dermatology, Ziekenhuisgroep Twente, Hengelo, The Netherlands; 3 Department of Biomedical Data Sciences, Leiden University Medical Center, Leiden, The Netherlands; 4 Department of Biomechanical Engineering, Delft University of Technology, Delft, The Netherlands; 5 Department of Dermatology, Roosevelt Kliniek, Leiden, The Netherlands

**Keywords:** Anatomy, Medical research, Preclinical research

## Abstract

Mechanical stress influences scarring of a surgical wound. Several lines have been proposed for the best excision direction. It is unknown if these lines still apply when the body posture changes. The objective is to measure the skin reaction force in four directions and determine the direction of least force. Secondary objective is to determine if the reaction force varies in a different body posture. Skin reaction force was measured with the compressiometer in 30 participants on four different locations (forearm/upper arm/shoulder blade/lower back) in four directions (0°–45°–90°–135°) and two body postures. The direction of least skin reaction force changed with a different body posture and was significant for the forearm (*p* < 0.01) and shoulder blade (*p* = 0.05) The skin reaction force in all four direction changed significantly in a different body posture, except the 45° line in the upper arm and shoulder blade. Our results demonstrate that the skin reaction force in four directions in four locations varies with change in body posture. Focus should therefore not only lay on choosing the right direction, but also on managing skin tension postoperatively.

## Introduction

Wound healing is a complex process and, its impairment, results in pathological scarring (i.e., hypertrophic, widened, atrophic, or keloid scarring^1^. Pathological scars are not only aesthetically displeasing, but also cause discomfort such as pain and itchiness and even limit mobility at certain anatomical locations^2^. The classic wound healing process comprises of three phases: hemostasis, inflammation and proliferation, and remodeling. A disruption in one of these phases may lead to delayed or impaired wound healing. Factors that negatively influence the cellular and molecular mechanisms involved include body weight, older age, hypoxia, and anatomical locations such as the shoulder area^3–5^. From a biomechanical perspective, mechanical stress applied on wounds is an important factor known to result in hypertrophic scars through mechanotransduction^6,7^: as mechanical force is converted into electrochemical activity by cells, alterations in the mechanical force influence wound healing. Studies in mouse models have shown that mechanical stress induces hypertrophic scar formation. It is suggested that multiple cytokines and pathways are involved in scar formation (including TGF-β overexpression, the Wnt signaling pathway, and the FAK-ERK-MCP1 pathway), as a result of mechanotransduction in wound healing^8^.

To avoid excessive mechanical stress on wounds, physicians should outline the prospective scars in recommended excision lines for skin surgery. The most commonly used excision lines are the Langer’s lines or Relaxed Skin Tension Lines (RSTL)^9^. These excisional lines are partly based on a static body posture in the anatomical position. Langer punched circular holes in cadavers in his study and demonstrated that the shape of the holes changed, indicating the direction of least tension. Kraissl used wrinkles visible in the human face, which are usually perpendicular to muscle action, to illustrate the direction of excision. Lines described by Borges (RSTL) follow furrows produced by pinching the skin when the skin is relaxed. Paul determined the direction of least tenasion using a novel tensiometer technique to examine preoperative- and perioperative skin. He quantified and compared his Biodynamic Excisional Skin Tension (BEST) Lines with the previously described Langer’s lines and RSTL. By placing a tensiometer in a wound in different directions (in total 360°), he measured the direction of least tension^10^. However, in daily life, the body moves continuously, and thus skin tension changes constantly^11^. The magnitude of its change differs per individual and per anatomical location; for example, a professional athlete likely demonstrates more variation in skin tension than a desk clerk. Moreover, knowledge about the variation in skin tension in different body postures and its implications for preferred excision lines remain limited.

This study investigated the effects of body postures on the skin reaction force in different directions using a recently developed compressometer^10^ to take measurements at four body locations in two different body postures.

## Materials and methods

### Hardware

**Figure 1:**
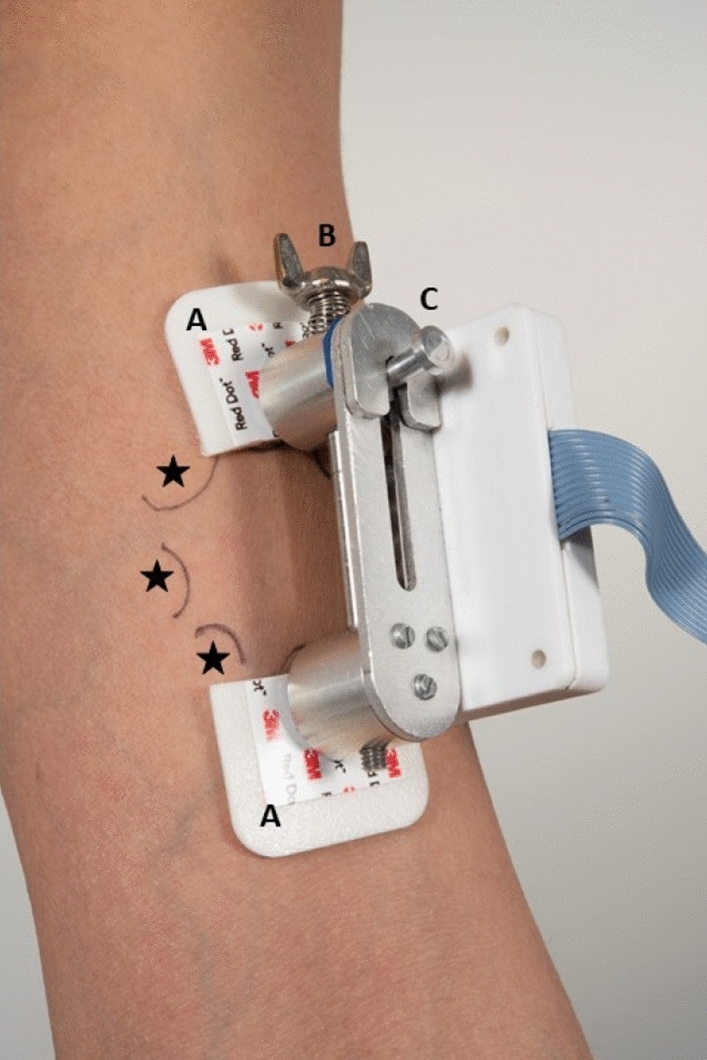
The compressiometer. (**A**) 3 M ECG stickers to fixate the device to the skin; (**B**) the compressed spring; (**C**) the button to release the spring; Asterisks: the locations for the 45, 90 and 135° measurements.

This study used a novel compressiometer (Fig. 1) recently developed and validated by the authors^12^. The device was used to determine the reaction force of the skin by determining the displacement of two fixed points on the skin under unidirectional loading. This reaction force is the result of both compression and extension of the skin when the fixed points are moved towards each other.

The device comprises a small measurement unit with two contact points that are attached on the skin using 3 M Red Dot ECG electrodes. The initial distance between the contact points is 30 mm. After placement, a button is pressed to unlock the locking mechanism. A compressed spring with known properties now drives the contact points towards each other until a new equilibrium is created between contact point movement and skin resistance. The displacement is measured in millimeters and is used to calculate the tension on the skin, as the spring properties are known.

### Clinical study

This study was registered in the Dutch Trial Register (trialregister.nl; trial ID NL8476; registration date 19-03-2020) and was approved by the Medical and Ethics Committee of the Leiden University Medical Centre. All experiments were performed in accordance with the relevant guidelines and regulations.

Participants were recruited through social media. Inclusion criteria were: 18–30-year-old, healthy individuals. Exclusion criteria were as follows: (1) physical movement impairment; (2) visible skin disease, scar, wound, or damaged skin on the area to be tested; (3) any connective tissue disease; and (4) a contact allergy to glue (used in electrodes).

**Figure 2:**
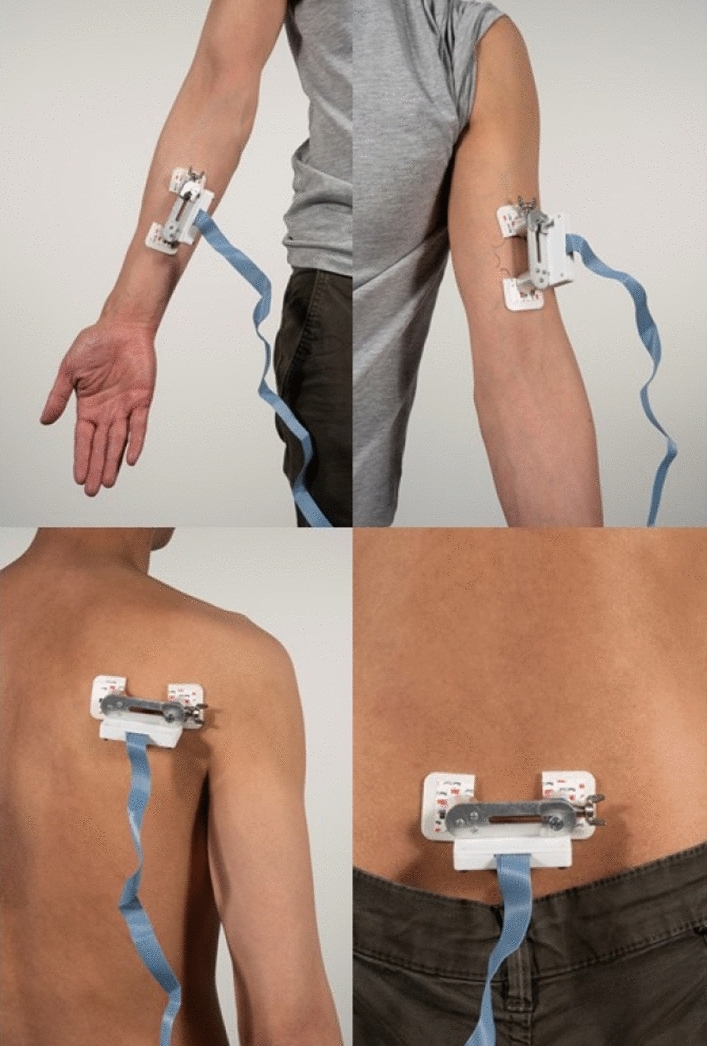
The postures: top left forearm, top right upper arm, bottom left shoulder blad, bottom right lower back.

**Figure 3:**
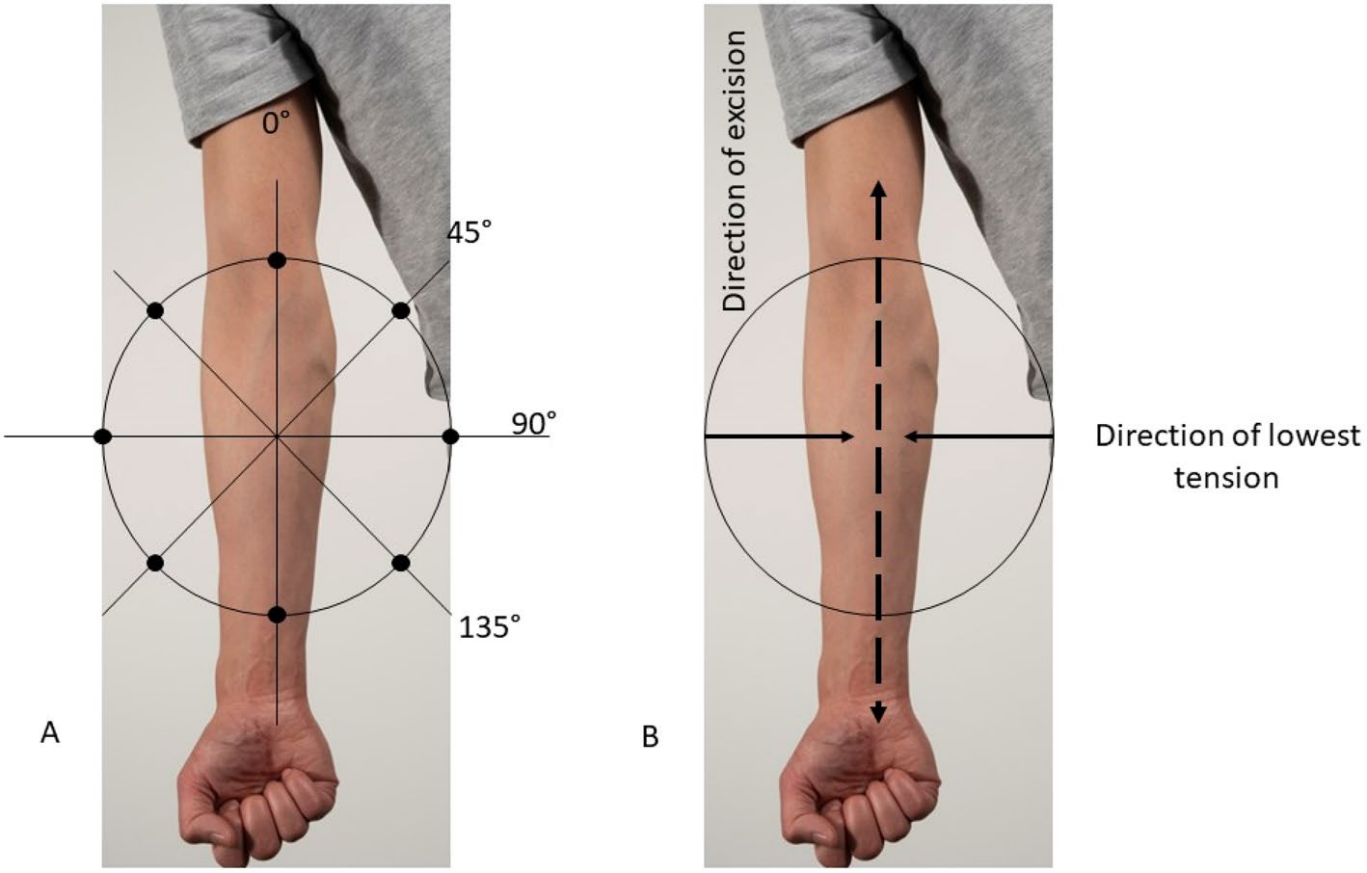
(**A**) The four angles measured: 0, 45, 90 and 135°. The 0 line represents the known Langer line from the literature. In theory, the 90° line should be the direction of lowest skin tension. (**B**) Example: if 90° is the lowest measured tension, the preferred excision line would be 90° perpendicular.

A standardized test protocol was conducted for each participant. Measurements were taken in two different postures at four different locations: (1) forearm: extension and flexion, (2) upper arm: extension and flexion, (3) shoulder blades: arms closed and arms wide with complete extension of the elbows, and (4) lower back: sitting up straight and sitting with the lower back completely bent (Fig. 2). The same procedure was used for each location and posture. With a premade plastic mold, lines were drawn for the 3 M electrodes at each location to ensure that measurements were taken in the same directions when the body posture changed. Two 3 M stickers were set in place, and the device was attached to the stickers. Finally, the spring was released, and the measurement was recorded. The four different directions always started corresponding with the 0° line according to the known Langer’s lines described in the literature. This line was used as a standardized starting point, as it is the most known and well documented line. From this starting point, the direction was changed in increments of 45° with 135° being the last angle at which measurements were taken (Fig. 3A). In this study, we chose the direction of least tension as the preferred direction of closure, rounding off to multiples of 45°. The same principle is applied for RSTL by Borges: the direction of excision is determined by pinching the skin with the forefinger and thumb. Thus the actual excision line is perpendicular to the direction identified to be associated with the lowest reaction force (Fig. 3B).

### Statistical analyses

The frequencies of the angles with the lowest skin tension were determined per location. To examine whether the direction of lowest reaction force differed between postures, we performed a variant of the McNemar–Bowker test that can deal with sparse tables (Edelmann and Goeman, 2022, A Regression Perspective on Generalized Distance Covariance and the Hilbert–Schmidt Independence Criterion, to appear in Statistical Science; https://imstat.org/journals-and-publications/statistical-science/statistical-science-future-papers/). We used the exact non-parametric method for calculating the *p*-value for this test, using 500,000 permutations.

The directions with the highest frequency were then used, and the mean reaction force (in Newtons) between two body postures was compared using paired t-tests. The same statistical analysis was performed with the mean reaction force for every direction separately to compare changes in the direction lowest reaction force between two body positions.

Statistical analysis was performed using SPSS statistics (IBM Corp., released 2020, IBM SPSS Statistics for Windows, Version 27.0. Armonk, NY: IBM Corp.) and R (R development Core Team, released 2017, Version 4.1.1, Vienna: R Foundation for Statistical Computing). Differences were considered to be statistically significant if *p* < 0.05.

## Results

In total, 33 individuals provided written informed consent for inclusion in this study; however, Three patients were excluded from analysis owing to measurement problems. The mean age of the remaining participants (28 females and 2 male) was 23.9 ± 2.5 years. There were no adverse reactions reported during measurements and no missing data. The frequencies of the angle with the lowest tension per location are shown in Table 1.

**Table 1:** Shows the frequencies and percentages of the lowest measured tension per angle.

	Angle (%)			
	0°	45°	90° (expected direction for Langer line)	135°
**Forearm**				
Extension	3 (10)	2 (7)	**24 (80)**	1 (3)
Flexion	6 (20)	**19 (63)**	5 (17)	0 (0)
**Upper arm**				
Extension	9 (30)	**15 (50)**	3 (10)	3 (10)
Flexion	10 (33)	**19 (63)**	0 (0)	1 (3)
**Shoulder blade**				
Arms closed	5 (17)	**11 (37)**	10 (33)	4 (13)
Arms wide	1 (3)	1 (3)	13 (43)	**15 (50)**
**Lower back**				
Sitting	**21 (70)**	5 (17)	2 (6.5)	2 (6.5)
Bend	**26 (87)**	1 (3)	1 (3)	2 (7)

In bold are the highest frequencies.

**Figure 4:**
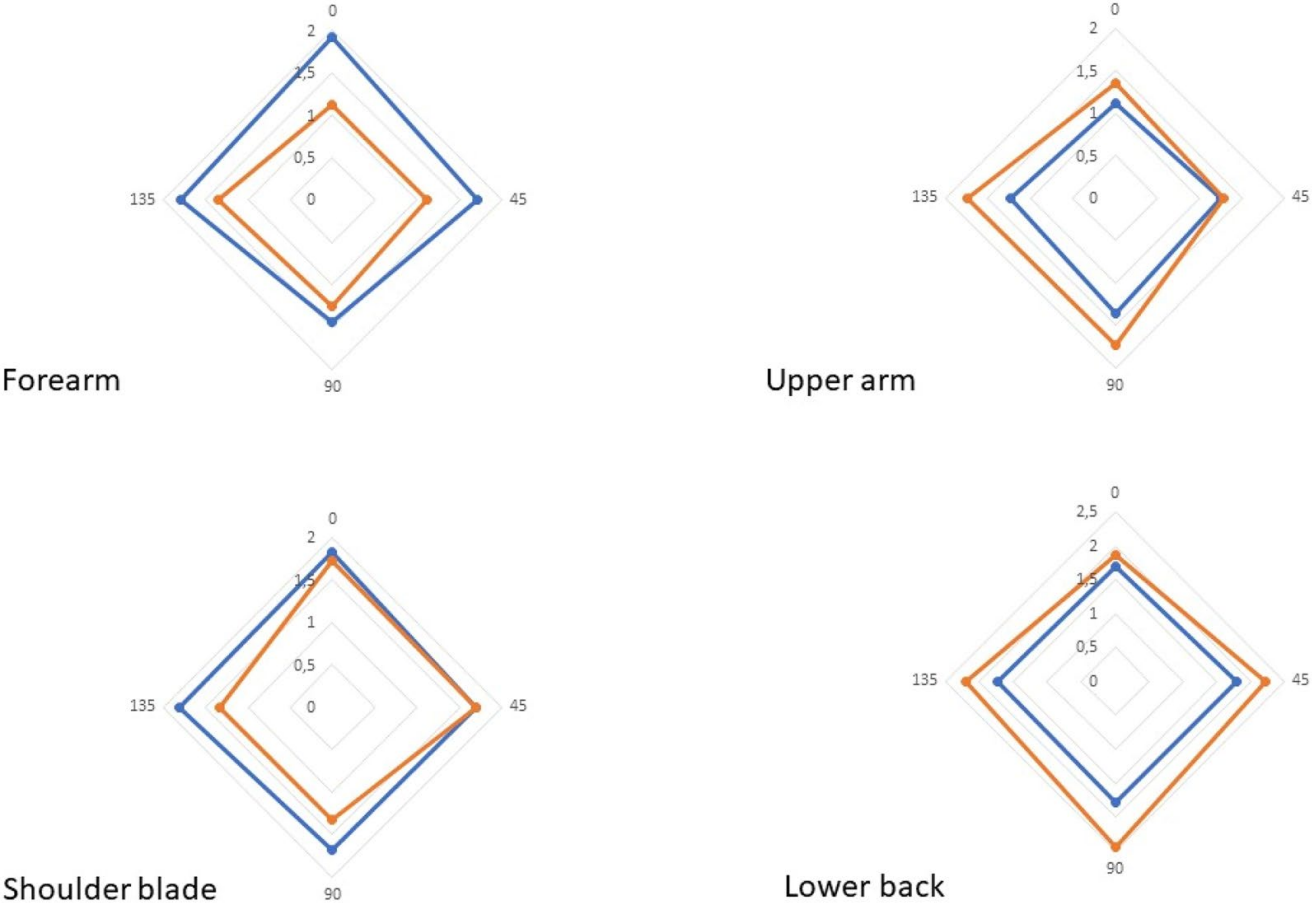
Shows the means of the lowest skin reaction force in Newton per direction (0, 45, 90 and 135°) in two body postures (blue = extension forearm and upper arm, arms closed shoulder blade and sitting straight lower back; orange = flexion forearm and upper arm, arms wide shoulder blade and sitting bend lower back).

The differences in skin reaction force in the four directions between the two body postures (e.g. flexion–extension, arms closed-arms wide, sitting-bent) are shown in Fig. 4.

### Skin reaction force in four directions

For the forearm, the mean reaction force (in the direction of lowest tension) for the forearm was 1.4 ± 0.29 N in extension and 1.0 ± 0.17 N in flexion (*p* < 0.01). The reaction force at 0°, 45°, 90° and 135° during extension and flexion was 1.9 ± 0.24 versus 1.1 ± 0.26 N (p < 0.01); 1.7 ± 0.27 versus 1.1 ± 0.21 N (p < 0.01); 1.4 ± 0.33 versus 1.2 ± 0.25 N (p < 0.01), and 1.8 ± 0.25 versus 1.4 ± 0.27 N (p < 0.01), respectively. For the upper arm, the mean reaction force (in the direction of lowest tension) was 1.16 ± 0.18 N in extension and 1.21 ± 0.21 N in flexion (*p* = 0.04). The reaction force at 0°, 45°, 90° and 135° during extension and flexion was: 1.1 ± 0.21 N versus 1.4 ± 0.25 N (p < 0.01); 1.2 ± 0.25 versus 1.3 ± 0.28 N (p = 0.419); 1.4 ± 0.27 versus 1.7 ± 0.18 N (p < 0.01); and 1.2 ± 0.18 versus 1.7 ± 0.22 N (p < 0.01), respectively. For the shoulder blade, the mean reaction force (in the direction of lowest tension) was 1.6 ± 0.23 N with arms closed and 1.3 ± 0.25 N with arms wide open (*p* < 0.01). The reaction force at 0°, 45°, 90° and 135° for the postures arms closed and arms wide open was 1.8 ± 0.19 versus 1.7 ± 0.24 N (p = 0.01); 1.7 ± 0.21 versus 1.7 ± 0.25 N (p = 0.975); 1.7 ± 0.25 versus 1.3 ± 0.27 N (p < 0.01), and 1.8 ± 0.27 versus 1.3 ± 0.29 N (p < 0.01), respectively. For the lower back, the mean reaction force (in the direction of lowest tension) was 1.5 ± 0.49 N while sitting straight and 1.9 ± 0.45 N while sitting bent (*p* < 0.01). The reaction force at 0°, 45°, 90° and 135° while sitting straight and sitting bent over was: 1.7 ± 0.25 versus 1.9 ± 0.26 N (p < 0.01); 1.8 ± 0.27 versus 2.2 ± 0.24 N (p < 0.01); 1.8 ± 0.16 versus 2.5 ± 0.22 N (p < 0.01), and 1.7 ± 0.24 versus 2.2 ± 0.22 N (p < 0.01), respectively.

### Direction of lowest skin reaction force

In body posture one, the direction of lowest skin reaction force with the highest frequency for the forearm (extension), upper arm (extension), shoulder blade (arms closed), and lower back (sitting straight) was 90° (80% of the measurements), 45° (50%), 45° (37%) and 0° (70%), respectively. In body posture number two, the direction of lowest skin reaction force with the highest frequency for the forearm (flexion), upper arm (flexion), shoulder blade (arms wide), and lower back (sitting bent) was: 45° (in 63% of the measurements), 45° (63%), 135° (50%) and 0° (87%), respectively. The difference in the direction of lowest skin reaction force due to change in body posture was significant for the forearm (*p* < 0.01) and shoulder blade (*p* = 0.05) but not significant for the upper arm and lower back.

## Discussion

In this study, we measured the skin reaction force at four locations in four directions to identify the direction of preferred compression (i.e. wound closure) and measured it in two different body postures to determine whether the reaction force increased or decreased. Our findings showed that the reaction force significantly increased or decreased when the body posture changed. Moreover, the direction of lowest reaction force changed between body postures. The preferred direction changes in different body postures were not always consistent with current recommendations such as Langer’s Lines. These results correspond with the data from a previous pilot study that also indicated a relationship between body posture and measured reaction force^12^.

The prevention of pathological scar tissue formation after (skin) surgery remains challenging. Current anti-scarring recommendation highlight the significant effect of mechanical tension on scar formation^13^. However, most recommendations are based on postoperative clinical studies and do not emphasize the magnitude of mechanical force for different body postures. Therefore, analyzing and measuring skin reaction force may aid in developing scar-reducing strategies.

Notably, our study did not use in vivo excision wounds to measure skin tension; however, we believe that our results are comparable to previous findings: the compression used imitates wound suturing and resembles the effect of excision lines^14^. In clinical practice, as physicians, we start with intact skin and must plan the ideal excision line exerting the lowest reaction forces on the scar. There are numerous recommendations for identifying the ideal excision lines in (skin) surgery, such as Langer’s lines, Kraissl’s lines, RSTL and BEST Lines^9,15–18^. In the present study, the only location with a finding consistent with the traditionally used Langer’s laines was the forearm in extension; all other findings indicated directions that were not in agreement with Langer’s lines. A comparison with the map of the more recent BEST lines by Paul et al. shows that the BEST lines are vertical on the upper and lower arm, whereas in the present study, the excision line with the lowest (perpendicular) force for the upper arm and forearm is at an angle. Moreover, for the lower back, our results recommended a vertical excision line instead of a horizontal line. However, this discrepancy is, attributable to the location of measurement, as our device was placed in the exact center. If our device were placed on a side of the lower back, the findings could have been more comparable to the BEST lines. The key difference in comparison with the previously described excision lines is that our findings varied with a change in body posture.

The human body is continuously in motion and subsequently skin tension keeps changing. Skaria proposes to take into account factors such as physical activity and gravity^11^. According to Skaria, in the case of cutaneous breast surgery, excision lines should be adapted to the shape of the individual patient’s breast^19^. Our findings support the hypothesis that alterations in body posture change the preferred excision line, as signified by the change in the reaction force visible on the forearm and shoulder blade. On the forearm, the direction changed from 90° to 45° as the body posture changed, whereas on the shoulder blade, the direction changed from 45° to 135°. These findings demonstrate that although it is difficult to determine the precise direction of lowest tension for these locations, extension of this research with more participants and validation of these findings using actual excisions may facilitate the development of a universal excision line body map. There is more certainty regarding excision lines for the lower back and upper arm, as these directions did not change significantly between postures.

Another important finding in the present study was that the skin reaction force in four directions at all studied locations changed significantly with a change in body posture, suggesting the presence of a continuous strain on the wound from these directions. Thus, reducing this strain postoperatively may be a strategy to prevent pathological scar formation^20^. This notion is consistent with previously reported clinical findings suggesting that postoperative tension-relieving therapies help in preventing pathological scarring^21–24^. A review by O’Reilly et al. indicates that using non-stretch tape on scars postoperatively is effective in reducing scar height, color, and itch (this applies only to surgical scars and not burn scars)^25^. Another study by Longaker et al. shows that using the commercially available Embrace device (Neodyne Biosciences) postoperatively reduces scarring^26^. This device comprises a silicone elastomeric dressing which can be prestrained before application. However, these studies are not supported by quantitative data on skin tension in various postures. In such studies, measuring the direction of taping using the novel device described in the present study may facilitate further optimization of tension relief.

### Limitations

The following study limitations must be noted: 1. Our study population was rather homogeneously young with a low mean age. However, older individuals exhibit more laxity in the skin (owing to reduction in collagen), and less tension is required to close large wounds^27^. 2. Our conclusion only applies to four specific anatomical locations with two body postures. Other anatomical locations need to be studies. 3. Our study used healthy, intact skin to measure skin tension. In future studies, we intend to measure skin tension in skin defects to compare preoperative and perioperative outcomes. The newly developed compressiometer is not suitable for intraoperative use.

Our study suggests that the recommended excision lines described in the literature are not always applicable owing to changes in body position. Furthermore, there remains continuous strain on the skin. In the clinical setting, it is not always feasible to choose the recommended excision line because of a certain anatomical location (e.g., in knee surgery^28^). Therefore, to help wound healing and reduce skin tension, postoperative scar reduction strategy (e.g., taping) should be considered. Future research focused on this reduction, for example, the use and duration, may facilitate further development of novel strategies.

## Conclusion

Our results demonstrate that the magnitude and direction of lowest skin reaction force vary with changes in body posture, making it more challenging to identify a single direction as the ideal direction of excision. The findings also suggests that it is relevant to measure the changes in tension; therefore, strategies to reduce mechanical stress on wounds to prevent pathological scar formation should focus not only on the direction of the scar, but also on postoperative tension reduction.

## Data Availability

The data that support the findings of this study are available from the corresponding author upon request.
